# Full-length transcriptome sequencing provides insights into the evolution of apocarotenoid biosynthesis in *Crocus sativus*

**DOI:** 10.1016/j.csbj.2020.03.022

**Published:** 2020-03-26

**Authors:** Junyang Yue, Ran Wang, Xiaojing Ma, Jiayi Liu, Xiaohui Lu, Sambhaji Balaso Thakar, Ning An, Jia Liu, Enhua Xia, Yongsheng Liu

**Affiliations:** a School of Food and Biological Engineering, Hefei University of Technology, Hefei 230009, China; b School of Computer and Information, Hefei University of Technology, Hefei 230009, China; c State Key Laboratory of Tea Plant Biology and School of Horticulture, Anhui Agricultural University, Hefei 230036, China; d College of Information Technology, Jiaxing Vocational Technical College, Jiaxing 314000, China; e Department of Biotechnology, Shivaji University, Kolhapur 416003, India; f Chongqing Key Laboratory of Economic Plant Biotechnology, College of Landscape Architecture and Life Science, Institute of Special Plants, Chongqing University of Arts and Sciences, Chongqing 402160, China; g Ministry of Education Key Laboratory for Bio-resource and Eco-environment, College of Life Science, State Key Laboratory of Hydraulics and Mountain River Engineering, Sichuan University, Chengdu 610064, China

**Keywords:** *Crocus sativus*, Saffron quality, Comparative transcriptomics, Apocarotenoid biosynthesis, Single molecular real-time (SMRT) sequencing

## Abstract

*Crocus sativus*, containing remarkably amounts of crocin, picrocrocin and safranal, is the source of saffron with tremendous medicinal, economic and cultural importance. Here, we present a high-quality full-length transcriptome of the sterile triploid *C. sativus*, using the PacBio SMRT sequencing technology. This yields 31,755 high-confidence predictions of protein-coding genes, with 50.1% forming paralogous gene pairs. Analysis on distribution of *Ks* values suggests that the current genome of *C. sativus* is probably a product resulting from at least two rounds of whole-genome duplication (WGD) events occurred at ~28 and ~114 million years ago (Mya), respectively. We provide evidence demonstrating that the recent β WGD event confers a major impact on family expansion of secondary metabolite genes, possibly leading to an enhanced accumulation of three distinct compounds: crocin, picrocrocin and safranal. Phylogenetic analysis unravels that the founding member (CCD2) of CCD enzymes necessary for the biosynthesis of apocarotenoids in *C. sativus* might be evolved from the CCD1 family via the β WGD event. Based on the gene expression profiling, CCD2 is found to be expressed at an extremely high level in the stigma. These findings may shed lights on further genomic refinement of the characteristic biosynthesis pathways and promote germplasm utilization for the improvement of saffron quality.

## Graphical abstract

**Figure:**
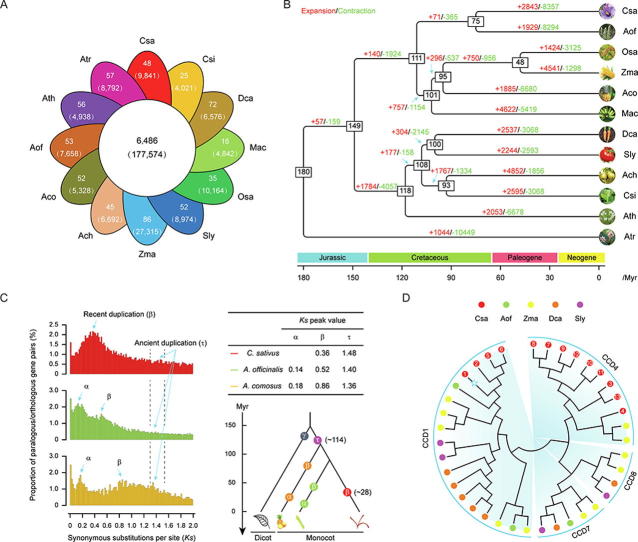

## 1 Introduction

*Crocus sativus* L. is a perennial flowering herb from the Iridaceae family. It is well known for its thread-like red stigmas with the commercial name ‘saffron’, which has been extensively used in food, coloration and medicine industries for thousands of years [1]. The earliest record of saffron (*Crocus* spp.) cultivation could be dated from about 2300 BC [2]. The current cultivars of *C. sativus* are sterile triploid with three homologous sets of chromosomes (2n = 3x = 24) that has been thought to be casually mutated from a diploid ancestor *C. carthwrightianus* [3], [4], [5] and domesticated in Greece beginning at around 700 BC [6]. The triploid genetic characteristics have been maintained by vegetative corms, which is also the major limitation for its genetic improvement. Since then, *C. sativus* cultivation was propagated throughout most Eurasia areas around the Mediterranean Sea and subsequently brought to North Africa, North America, and Oceania [7]. Nowadays, the most important countries for *C. sativus* breeding and saffron industries include Iran, Spain, India, Greece, Azerbaijan, Morocco and Italy [8].

Impressively, its flowers start to open in October with a strong pleasant sweet smell. Biochemical studies have shown that the flowers, especially the red stigmas, could accumulate a rich source of volatile and nonvolatile components that confer characteristic color, aroma and flavor to saffron [9]. The crocin is the typical egg-yolk yellow color producer, while safranal and picrocrocin are responsible for endowing the stigmas with hay-like aroma and spicy flavor, respectively [10]. These three kinds of apocarotenoids harmonize to make saffron an irreplaceable ingredient in the kitchen. Furthermore, the significant amounts of apocarotenoids have also been highly reputed in alleviating various ailments, such as cramps, depression, anxiety, cardiovascular diseases, nervous disorders and cancer [11], [12], [13].

Apocarotenoids, a class of carotenoid derivatives, are preferentially accumulated in stigma tissue of *C. sativus* with a maximum level at fully-developed scarlet stage [14]. Therefore, their biosyntheses should be developmentally regulated across the life span. In higher plants, carotenoid-related genes have been extensively studied through both forward and reverse genetic approaches [15]. But in *C. sativus*, only several structural genes, such as aldehyde dehydrogenase (ALDH), carotenoid cleavage dioxygenase (CCD), glucosyltransferase (GT), phytoene synthase (PSY) and uridine diphosphate glycosyltransferase (UGT) have been characterized so far [16], [17], [18], [19], [20], [21], [22]. The majority of genes that involved in the apocarotenoid biosynthesis are not yet known.

Previously, the second generation sequencing (SGS) technology has been intensively performed for discovery of functional genes by detecting their expression levels. Initially, through the utilization of 454 pyrosequencing for stigma expressed transcripts at six developmental stages, a novel CCD member that catalyzes the first dedicated step in crocin biosynthesis was identified and characterized [19]. Later, transcriptome dynamic analyses were employed to investigate different tissues of *C. sativus* to gain insights into structural genes and transcription factors involved in regulation of apocarotenoid biosynthesis [23], [24], [25], [26]. Another investigation by Malik and Ashraf focused on exploring the family members and expression patterns of zinc-finger transcription factors based on their previous SGS data [23], [27]. Although remarkable success has been achieved with the SGS technology, the inability to obtain full-length transcripts due to the limitation of read length is still a major challenge, which has hampered not only the whole genome assembly but also the individual gene isolation [28]. Comparatively, the third generation sequencing (TGS) technology, also known as single-molecule real-time (SMRT) sequencing, could yield kilobase sized sequence reads that are usually sufficient to represent full-length mRNA molecules [29]. Specially, SMRT sequencing developed by Pacific Biosciences (PacBio, CA, USA) is able to provide sequence reads with an average length exceeding 10 Kb in a single run (http://www.pacb.com/smrt-science/smrt-sequencing/read-lengths/). Meanwhile, PacBio has rationally designed an integrated pipeline SMRT Analysis software suite (version 5.1.0; https://www.pacb.com/support/software-downloads/) to effectively reduce error rates accumulatively caused by SMRT sequencing. After self-correction via circular consensus sequence (CCS) reads, the error rates of SMRT sequencing are expected to be less than 1% [30]. In plants, PacBio SMRT sequencing has been progressively utilized in functional gene annotation and alternative splicing identification [31], [32], [33], [34], [35]. Based on these advantages, SMRT sequencing has also been intriguingly employed to characterize the flowering gene regulatory network in *C. sativus* [36].

In the present study, we have adapted the PacBio SMRT sequencing to generate full-length transcriptome of *C. sativus* derived from the entire plant including five typical tissues: corm, leaf, tepal, stamen and stigma. Since no genome sequence is currently available due to the complex polyploidy and high heterozygosity in *C. sativus*, our full-length transcriptomic data could be alternatively allowed for documenting the genomic signatures in some cases, which will not only significantly improve the sequence integrity and functional annotation of putative genes, but also provide novel insights into the evolutionary status of *C. sativus* in general as well as apocarotenoid biosynthesis in particular.

## 2 Materials and methods

### 2.1 Plant material and growth condition

*C. sativus* L. was grown under natural conditions in an open farmland from November to May of the following year, and then transplanted into a house for cultivation until flowering. Our experimental farms are situated at Jiaxing, Zhejiang province, People's Republic of China (N30°39′, E120°42′). The annual average temperature and precipitation are recorded as 15.9 °C and 1168.6 mm, respectively. At flowering time (on Nov 11th, 2017), three individual plants including healthy corm, leaf, tepal, stamen and stigma were randomly collected for biological replicates. After collection, these tissue samples were immediately placed in a cryonic chamber with liquid nitrogen and then preserved at −80 °C for storage.

### 2.2 Library preparation and PacBio sequencing

Total RNAs were separately extracted from the three replicates of *C. sativus* samples using the TRIzol reagent (Invitrogen, MA, USA) by following the manufacturer's instructions. Equal RNA amounts from each extraction were pooled together for PacBio SMRT sequencing. The quality and quantity of the isolated RNAs were evaluated by Agilent RNA 6000 Nano Kit and 2100 Bioanalyzer instrument (Agilent Technologies, CA, USA). Only the qualified extractions with optical density ratios of λ_260/280_ (1.8–2.1) and λ_260/230_ (2.0–2.5) were chosen for the synthesis of cDNA molecules. Then, full-length cDNA strands were prepared by using the SMARTer PCR cDNA Synthesis Kit (Clontech, CA, USA) and size-selected with the Blue Pippin system (Sage Science, MA, USA). Subsequently, the cDNAs obtained were amplified to construct the SMRT libraries using the SMRTbell^TM^ Template Prep Kit (PacBio, CA, USA) according to user manual. Finally, a total of two SMRT cells were sequenced on the PacBio Sequel platform by Personal Biotech (Shanghai, China), with a 240-min collection protocol along with stage start.

### 2.3 SMRT processing and sequence clustering

The raw data produced by the PacBio Sequel sequencing were processed through the SMRT Analysis software suite (version 5.1.0). The standard protocols were used to remove adapters and artefacts for the generation of reads of insert (ROIs) sequences with parameters of full passes ≥ 0 and read quality > 0.8. Typically, ROIs were classified to four categories: full-length chimeric (FLC), full-length non-chimeric (FLNC), non-full-length (NFL) and short reads (≤300 bp). Only reads with both poly-A tail and two primers were defined as full-length categories in the present study. Then, FLNC reads were clustered into CCS reads using the Iterative Clustering for Error Correction (ICE) algorithm. Combined with NFL reads, these FLNC CCS reads were polished with the Quiver program [37]. High-quality transcripts with post-correction accuracy of >99% were retained for further analysis. Finally, redundant sequences in the high-quality transcripts were removed by the CD-HIT-EST program (version 4.6.1) [38] with a similarity of 0.90. The BUSCO assessment (version 3.0.2) [39] was employed to evaluate the integrity of the full-length transcripts without redundancy, and the number of embryophyta genes used in this evaluation was 1440.

### 2.4 Gene prediction and ncRNA identification

Putative genes as well as their protein-coding regions in *C. sativus* were predicted by using the TransDecoder software (version 5.2.0; https://github.com/TransDecoder/TransDecoder). Quality validation of the protein-coding genes was evaluated through the alignment with the expressed sequence tags (EST) and SGS transcriptomic data. For gene annotation, the BLASTP program (version 2.2.26) was conducted between the encoded proteins of *C. sativus* and a suite of protein databases, including the nr, Swiss-Prot, KEGG, and COG databases, with an *E*-value threshold of 1*e*-5. Within the alignments against each database, the best blast results were reserved. While resultant annotations from different databases conflict, a defined priority order of nr, Swiss-Prot, KEGG and COG was followed to determine the annotation entries. Subsequently, the Blast2GO local pipeline (version 3.2) [40] and WEGO online tool (version 2.0) [41] were performed to assign and compare the GO terms of gene products in turn. The motifs and domains within the encoded proteins were identified by the InterProScan software (version 5.29) [42] against multiple public databases. The transcription factors were predicted and classified by the iTAK online program [43]. The enrichment analysis of both GO terms and Pfam domains was performed using the Hypergenometric test as our previous description [44].

In addition, five different types of short non-coding RNA (ncRNA) genes, namely ribosomal RNA (rRNA), transfer RNA (tRNA), small nucleolar RNA (snoRNA), small nuclear RNA (snRNA) and microRNA (miRNA), were predicted by the INFERNAL software (version 1.1.2) [45] through homology search against the Rfam database (version 12.2) [46] with default parameters. The putative target genes of miRNAs were predicted by using the psRNATarget online program with default parameters [47]. Simple sequence repeats (SSRs), also known as microsatellites, were identified by the RepeatMasker package (version 2.6.0) [48] and counted by the MIcroSAtellite (MISA) Perl script (http://pgrc.ipk-gatersleben.de/misa/). The minimum repeat unit number for mononucleotide was set at 12, dinucleotide at 6, trinucleotide at 4, and at 3 for tetranucleotide, pentanucleotide as well as hexanucleotide.

### 2.5 Gene family and genome evolution

The OrthoFinder package (version 2.2.7) [49] was employed to identify gene families between *C. sativus* and 11 representative plant species, including *Actinidia chinensis* (http://kir.atcgn.com/) [50]*, Ananas comosus* (http://pineapple.angiosperms.org/pineapple/html) [51]*, Asparagus officinalis* (https://phytozome.jgi.doe.gov) [52]*, Arabidopsis thaliana* (https://www.arabidopsis.org) [53]*, Amborella trichopoda* (https://phytozome.jgi.doe.gov) [52]*, Camellia sinensis* (http://tpia.teaplant.org) [54]*, Daucus carota* (http://apiaceae.njau.edu.cn) [55]*, Musa acuminata* (https://banana-genome-hub.southgreen.fr/) [56]*, Oryza sativa* (https://rapdb.dna.affrc.go.jp/) [57]*, Solanum lycopersicum* (https://solgenomics.net) [58]*,* and *Zea mays* (https://www.maizegdb.org/) [59]. The species-specific genes as well as their belonging families were determined on the basis of the presence or absence in a given species. We investigated the dynamic evolution (expansion and contraction) of orthologous gene families using the latest version of Computational Analysis of gene Family Evolution (CAFE 3.1) [60] with probabilistic graphical models. Evolutionary relationships among these 12 plants were resolved by using the Randomized Accelerated Maximum Likelihood package (RAxML version 8) [61] based on 257 single-copy and high-quality orthologous genes. The phylogenetic trees obtained were visualized using the MEGA tool (version 10) [62]. The estimating divergence times were directly retrieved from the online TimeTree database [63].

By using the paralogous gene pairs, we aim to detect the WGD events occurred in a given species. Briefly, we firstly screened the paralogous gene pairs from the analyzed results produced by the OrthoFinder package (version 2.2.7) [49], yielding a total of 50,699, 39,898 and 85,615 gene pairs in the proteomes of *C. sativus*, *A. officinalis* and *A. comosus*, respectively. They separately represented approximately 50.1% (15,900 in 31,755), 47.5% (13,009 in 27,395) and 53.5% (14,447 in 27,024) of the total protein-coding genes. We then calculated the values of synonymous substitutions per synonymous site (*Ks*) for these gene pairs based on the NG (Nei & Gojoberi) method implemented in the PAML program (version 4.9) [64]. Finally, the *Ks* distribution for each species was plotted and displayed using R language (version 3.2.5). The peak *Ks* value was further converted to the divergence time by using the equation T = *Ks*/2λ, where λ is the substitution rate of 6.5 × 10^−9^ mutations per site per year [65]. The labelled name of each WGD event was referenced from published literature [66], [67].

### 2.6 Phylogenetic tree and expression pattern

We identified genes encoding CCD enzymes in our transcriptomic data of *C. sativus* as well as in the released genomic data of *A. officinalis*, *D. carota*, *S. lycopersicum* and *Z. mays*. Only those proteins with a length greater than 100 amino acids were retained for further analysis. In total, we obtained 38 protein sequences from the five representative plants. Subsequently, multiple sequence alignments of these CCD proteins were performed by the ClustalW tool (version 2.1) [68]. Finally, a maximum likelihood phylogenetic tree was constructed by the MEGA tool using the neighbor-joining (NJ) method (version 10) [62]. The bootstrap process was replicated 1000 times.

To calculate the expression pattern of the identified CCD genes in *C. sativus*, we first mapped the clean SGS RNA-Seq reads derived from five tissues (corm, leaf, tepal, stamen and stigma) to the defined 31,755 protein-coding genes using the Trinity software (version 2.6.6) [69] with default parameters, and then computed the Fragments Per Kilobase of transcript per Million fragments mapped (FPKM) values for each CCD gene with log_2_-transformed. The differentially expressed genes were identified via pair-wise comparisons of gene expression patterns between stigma and the other four tissues (corm, leaf, tepal and stamen) by the ‘DESeq’ package in R language (version 3.2.5). Here, the *P*-value threshold of <0.05 and a fold-change threshold of >2 were employed to define the significant differences. The raw SGS RNA-Seq reads were downloaded from the Gene Expression Omnibus under the accession number GSE65103. We visualized the gene expression pattern by using the ‘pheatmap’ package in R language (version 3.2.5).

## 3 Results and discussion

### 3.1 High-quality reads obtained from the single molecule sequencing-derived transcriptome

**Supplementary Fig. S1:**
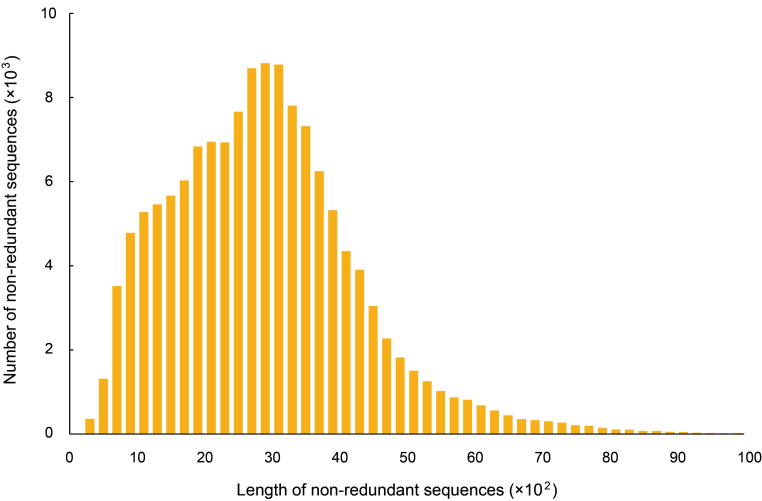
Length distribution of the obtained non-redundant sequences.

To obtain a representative full-length transcriptome for *C. sativus*, total RNAs extracted from the entire plant at full-bloom stage were sequenced on two SMRT cells using the PacBio Sequel system. This generated 1,133,474 reads of insert (ROIs) with a total of 9,514,218 subreads. Through the standard SMRT Link Analysis pipeline (version 5.1.0), 596,356 full-length non-chimeric (FLNC) reads (52.61%) with the complete transcripts region from 5′ to 3′ end were acquired (Supplementary Table 1). After error correction via the ICE algorithm and the Quiver program [37], we obtained 178,411 high-quality CCS reads (>99% accuracy). Then, the CD-HIT-EST package was employed to remove redundant sequences with a similarity of 0.90, consequently resulting in a number of 138,773 non-redundant sequences. The majority of these non-redundant sequences ranged from 800 to 4000 bp in size (Supplementary Fig. 1) Figure Supplementary Fig. S1, which is comparable to the length distribution of sequences generated by PacBio Iso-Seq sequencing [36]. By contract, two previous studies based on the SGS technology have respectively obtained unigenes with average length of 610 [23] and 1047 [24] bp, even after assembly. Hence, our results further demonstrate that SMRT sequencing is more advantageous over SGS technology in capturing the full-length transcripts of *C. sativus* [36].

**Fig. 1:**
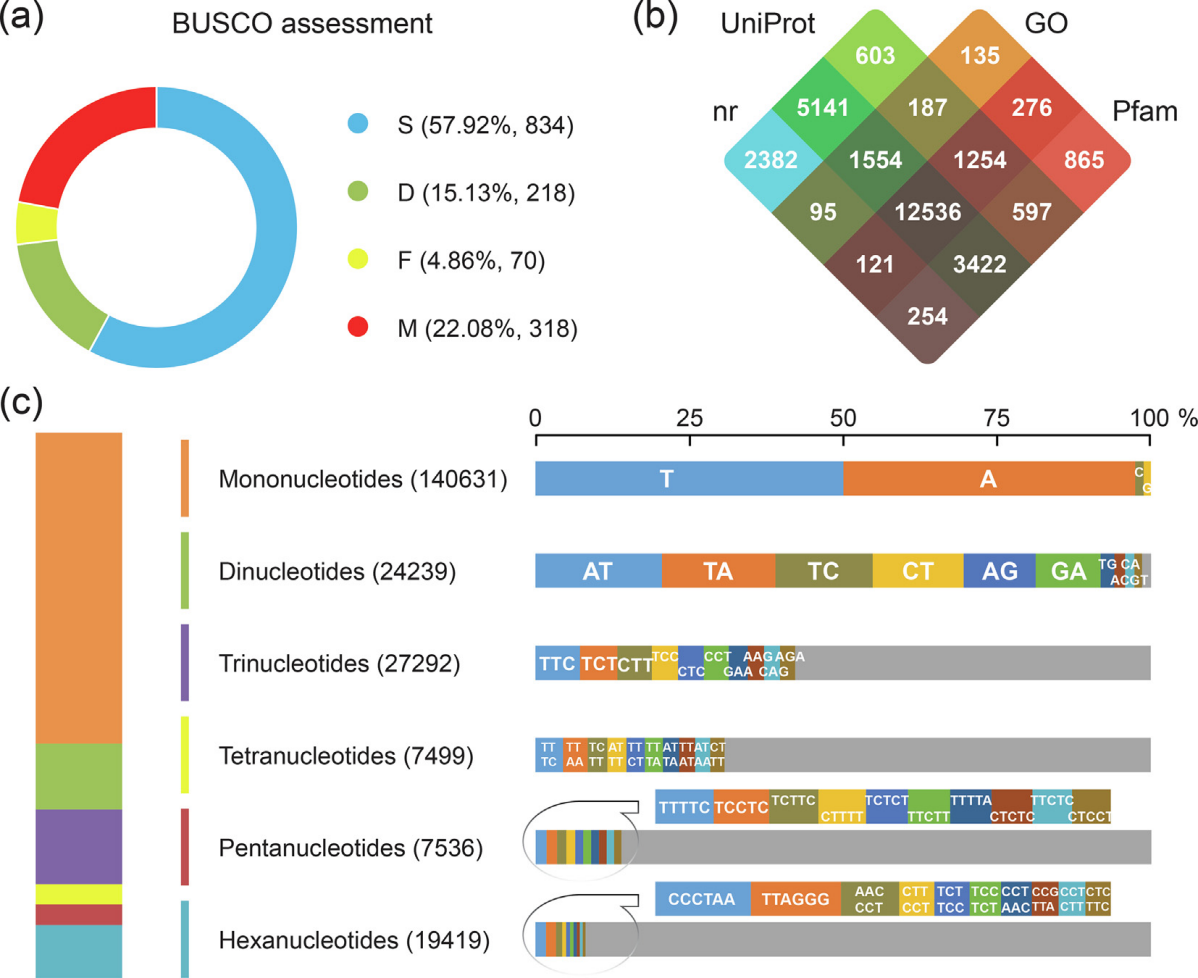
Summary of sequence quality and annotation for the full-length transcriptome in *C. sativus*. (a) Quality assessment with the BUSCO tool showed proportions classified as Complete and single-copy (S, blue), Complete and duplicated (D, green), Fragmented (F, yellow) and Missing (M, red). (b) The numbers of protein-coding genes annotated in the nr, UniProt, GO and Pfam databases were illustrated by Venn diagram. (c) Simple sequence repeats (SSRs) including six main classes were counted. Frequency of the top ten motifs (if any) in each SSR class was present. (For interpretation of the references to color in this figure legend, the reader is referred to the web version of this article.)

To further validate the sequence quality, we have firstly aligned all ESTs of *C. sativus* available in the GenBank database (on March 13, 2019) and obtained a mapping rate of 93.9% (*E*-value ≤ *e*-03 and identity ≥ 80%); secondly, the two SGS datasets also exhibited excellent alignments with the mapping rates of 82.93% [23] and 71.46% [24], respectively (*E*-value ≤ *e*-03 and identity ≥ 80%). In addition, quality assessment with the BUSCO tool [39] showed that complete sequences accounted for 73.06% (1052 in 1440) of the conserved core eukaryotic genes (Fig. 1A). The relatively high coverage of sequence mapping demonstrated a satisfying quality of our full-length transcripts by SMRT sequencing in this study. Since the genome sequence has not been fully deciphered, the current high-quality full-length transcripts were capable of identifying the complete coding regions of proteins and describing the evolutionary history of *C. sativus* [70].

In combination with *ab initio* prediction and homology search, we have defined 31,755 protein-coding genes with an average GC content of 48.66% in the CDS regions. The average and N50 length of the identified CDS sequences were 918 and 1131 bp, respectively. Among them, 30,197 (95.09%) could be transcriptionally supported by both of the SGS datasets [23], [24] and 29,422 (92.65%) could be functionally annotated to a suite of functional databases (Fig. 1B). In a previous investigation using SMRT sequencing, 64,562 sequences were annotated and accounted for 85.7% of the total unigenes [36], by contrast to the 58.5% (37,696) [23] and 54% (105,269) [24] derived from SGS technology. Our study and other investigations suggested that, over SGS technology, SMRT sequencing could not only produce comparatively longer sequences, but also provide improved annotation integrity of functional genes upon the transcriptomic data of *C. sativus*.

Among the gene-encoding proteins obtained, 1130 transcription factors (TFs) were identified and classified into 64 distinct families (Supplementary Table 2). The highest number of members was found for C3H family, followed by bHLH, bZIP and MYB-related families. Actually, the TFs involving zinc-finger motifs have been previously documented due to their potential biological functions related to the regulation of apocarotenoid biosynthesis [27]. A total of 81 zinc-finger TFs from the SGS data were identified and grouped into eight subfamilies, such as C2H2 (29) and C3H (20). In our study, 88 C3H and 56 C2H2 members were identified, accounting for ~ 13% of the total predicted TFs. Based on the statistics in plant transcription factor database [71], almost same percentage of zinc-finger TFs occurs across different plant species, covering up to approximately 16–18% out of the total TFs. Compared to the previous study [27], our results showed a higher coverage in annotating target sequences throughout the entire genome. This is not a surprise as the *de novo* assembly of short reads might be challenged by the repetitive sequences, such as the identical motifs or regions distributed among the same gene family [72], [73]. Apparently, the additional TFs identified in our study could be beneficial for the construction of full-scale gene regulatory network.

Sequence alignments were also carried out to predict short ncRNA genes, consequently yielding 821 rRNA genes, 393 tRNA genes, 38 snoRNA genes, 15 snRNA genes, and 10 miRNA genes (Supplementary Table 1). Among them, miRNA has been well demonstrated in negatively regulating gene expression at post-transcriptional or transcriptional level [74]. By aligning the obtained miRNAs with protein-coding genes, we identified a total of 645 candidate targets (Supplementary Table 3). Similar to other studies, a single type of miRNAs alone can bind multiple gene products [75], [76]. Interestingly, there were a relatively large number of the zinc-finger TFs among the candidate targets, showing a potential connection between the tested miRNAs and the regulation of apocarotenoid biosynthesis by the zinc-finger TFs [27]. On the contrary, we did not find apocarotenoid biosynthesis genes targeted by any miRNAs, suggesting miRNA may function mainly upon TFs to perform global regulation in *C. sativus* [77].

Finally, we annotated 226,616 SSRs distributed in 131,666 sequences, accounting for ~ 94.9% of the total transcripts (Fig. 1C). This high proportion of SSRs was resulted from a fairly large number of ‘A/T’ repeats (67,322, ~48.5%) in the mononucleotide class. Among them, only 3,714 SSRs in 3,381 CDS sequences (~10.6%) were identified, indicating that SSRs are more abundant in the non-coding regions than in the coding regions, which was commonly observed in both plants and animals [78], [79]. As one of the most useful molecular markers, SSRs can be easily detected by the standard PCR technology, which is quite suitable for studies on allopolyploid species [80]. Undoubtedly, our data would enrich the existing repository of SSR markers for genetic studies and breeding programs of the triploid *C. sativus*.

### 3.2 Whole-genome duplication events estimated from the comparative analysis of full-length transcripts

**Fig. 2:**
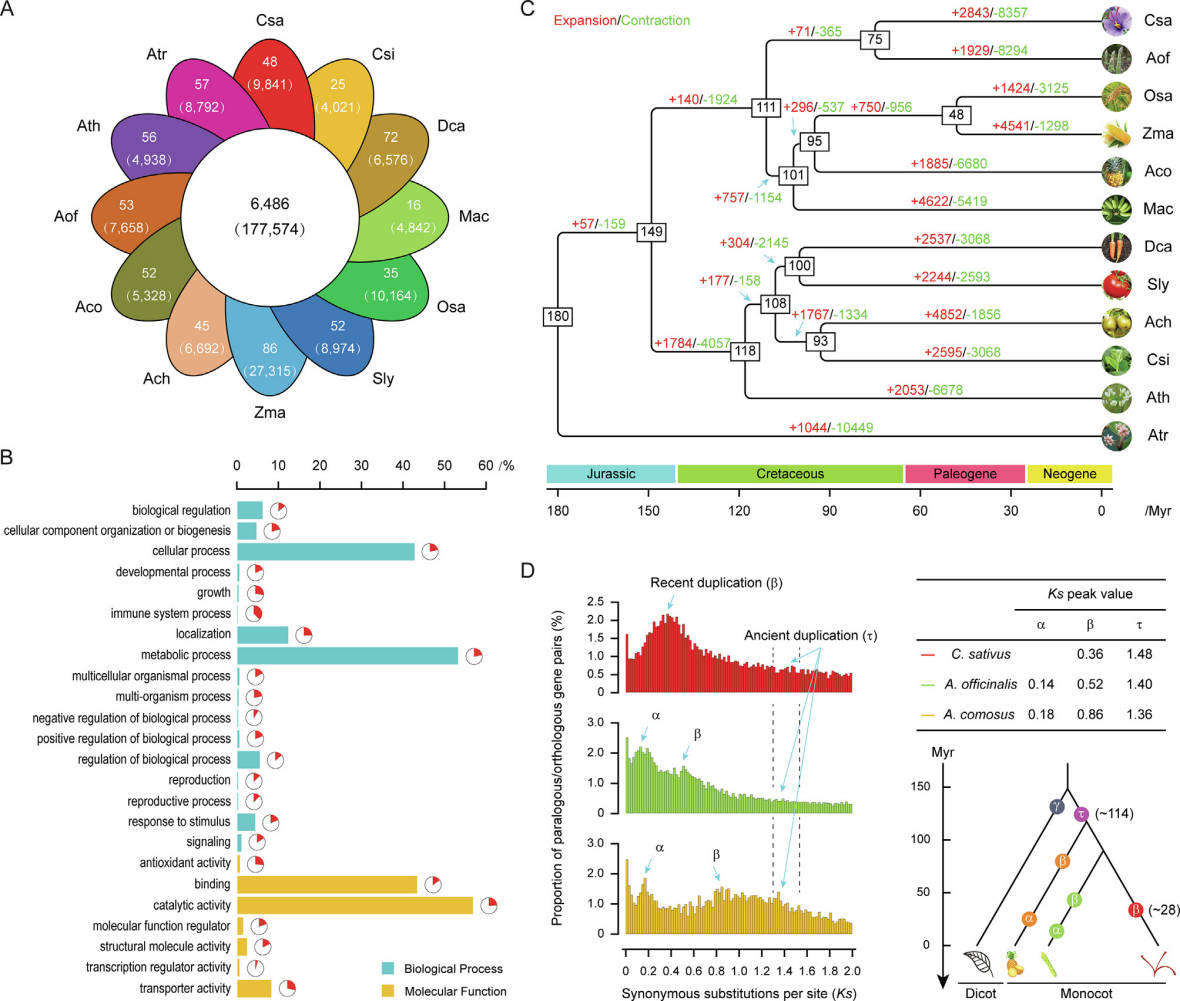
Comparative analysis of genome evolution and gene family in *C. sativus*. (a) Venn diagram showed the shared and specific gene families distributed among *C. sativus* and 11 representative plant species. Each value in parentheses represented the number of genes within corresponding families (without parentheses). Three-letter acronym for the abbreviation of each species name. (b) The specific genes in *C. sativus* were assigned to biological process and molecular function categories according to the GO annotation. Pie diagram next to each histogram bar represented the proportion of a given GO term in the specific genes to the proteomes of *C. sativus*. (c) Expansion and contraction of gene families among the 12 plant species. Phylogenetic tree was constructed based on 257 high-quality 1:1 single-copy orthologous genes using *A. trichopoda* as outgroup. The numerical values on each branch of the tree represented gene families undergoing gain (red) or loss (green) events. The number of gene families predicted in the most recent common ancestor (MRCA) was 19,106. The numerical values in the box denoted the estimated divergent time of each node (Myr). Three-letter acronym for the abbreviation of each species name. (d) Whole-genome duplication events detected in *C. sativus* as well as in *A. officinalis* and *A. comosus*. The occurrence time was estimated from the peak *Ks* value. (For interpretation of the references to color in this figure legend, the reader is referred to the web version of this article.)

To reveal the genomic foundation of species adaptation during evolution, we have compared the identified proteome of *C. sativus* with those of 11 representative plants, including *A. chinensis, A. comosus, A. officinalis, A. thaliana, A. trichopoda, C. sinensis, D. carota, M. acuminata, O. sativa, S. lycopersicum,* and *Z. mays*. Consequently, a total of 19,131 orthologous gene families comprising 311,825 genes were obtained. Of these, 177,574 genes belonging to 6,486 families were shared among all these 12 plants, representing a core set of ancestral clusters (Fig. 2A). On the other hand, 9841 genes in 48 different families were specific to *C. sativus*, suggesting their unique biological and phytochemical properties within the *Crocus* sublineage (Fig. 2A).

Functional enrichment analysis based on the gene ontology (GO) annotation revealed that the specific genes tend to possess ‘metabolic process’ (in ‘biological process’) and ‘catalytic activity’ (in ‘molecular function’) categories as summarized at the level 2, expanding our knowledge of metabolic network architecture in *C. sativus* (Fig. 2B). Among them, a certain number of biosynthetic pathways were related to the major saffron characteristic secondary metabolites (e.g., crocin, picrocrocin and safranal). The enriched GO terms included ‘oxidoreductase activity, acting on single donors with incorporation of molecular oxygen, incorporation of two atoms of oxygen’ (GO: 0016702, *P*-value < 0.001), ‘hydrolase activity, hydrolyzing O-glycosyl compounds’ (GO: 0004553, *P*-value < 0.001) and ‘carotenoid dioxygenase activity’ (GO: 0010436, *P*-value < 0.001) (Supplementary Table 4). PFAM annotation further verified that genes involved in the biosynthesis of apocarotenoids were significantly enriched in ‘retinal pigment epithelial membrane protein superfamily and carotenoid oxygenase family’ (PF03055, *P*-value < 0.001) (Supplementary Table 5), which was reported to encode enzymes associated with the cleavage of various carotenoids (e.g., phytoene, carotene, lycopene, and zeaxanthin) at different kinds of chemical bonds [81]. Remarkably, terpenoids represent a large and diverse class of natural products that contribute significantly to aromas, resins and essential oils [82]. In saffron, the strong-smelling volatiles include at least 34 terpenic components, such as terpenes, terpene alcohols and their esters [83]. Here, we found that the specific genes in *C. sativus* were also significantly enriched in biological functions related to terpenoid biosynthetic process (GO: 0016114, *P*-value < 0.05), which might motivate the biosynthesis of aroma volatiles unique to saffron (Supplementary Table 4).

In flowering plants, the expansion and/or contraction of gene families have been well documented as crucial driving forces in lineage splitting and function diversifying [84]. Here, we characterized gene families probably undergoing discernible change in adaptive evolution through divergent branches, with particular emphasis on those involved in plant traits and saffron qualities of *C. sativus*. Meanwhile, phylogenetic analysis was performed to reflect the evolutionary relationships among species as well as their estimated divergent times (Fig. 2C). Our results showed that, among the 19,106 gene families inferred to be present in the most recent common ancestor (MRCA) of the 12 representative plant species analyzed, 8357 families were contracted in *C. sativus*, whereas new gene copies were gained within 2843 families (Fig. 2C). GO annotation of 1792 genes from 212 families with significant expansions (*P*-value < 0.05) demonstrated that they were mainly enriched in functional categories related to electron transport chain (Supplementary Table 6), such as ‘phosphoenolpyruvate carboxykinase (ATP) activity’ (GO: 0004612, *P*-value < 0.001), ‘phosphoenolpyruvate carboxykinase activity’ (GO: 0004611, *P*-value < 0.001), ‘electron transporter’ (GO: 0045158, *P*-value < 0.001) and ‘respiratory electron transport chain’ (GO: 0022904, *P*-value < 0.001). The electron transport chain activities have been known to enable many metabolic processes, for example, the biosynthesis of aspartate [85], ascorbate [86], phytoene [87] and carotenoid [88]. Most likely, the expansion of gene families related to electron transport chain could also facilitate the biosynthesis of apocarotenoids via enhanced supplies of necessary precursor metabolites and energy in *C. sativus* [89].

Interestingly, genes containing the functional domain of male sterility proteins (PF07993, *P*-value < 0.001; PF03015, *P*-value < 0.05) were also found among the most highly enriched functional categories in the expanded families (Supplementary Table 7). These findings suggest a possible mechanism for evolution or domestication of this sterile triploid *C. sativus* promoted by either natural or artificial selection. In traditional agriculture practice, male sterility was known to spontaneously evolve and could be extensively used to produce offspring with compensatory advantages over their parents [90]. Thus, our observation of a large expansion occurred in functional genes related to male sterility may imply some kinds of selection pressures or adaptive responses targeted for desirable characteristics in *C. sativus*, such as increased production, improved quality, enhanced adaptation and genetic stability against various biotic and abiotic stresses. On the contrary, functional enrichment analysis of 881 genes within 316 significantly contracted families (*P*-value < 0.05) included a certain categories related to sexual reproduction, such as ‘recognition of pollen’ (GO: 0048544, *P*-value < 0.001; Supplementary Table 8) and ‘PAN-like domain’ (PF08276, *P*-value < 0.05; Supplementary Table 9). Coordinately, expansion or contraction of individual gene families could make synergistic effects in alleviating the costly consumption involving male reproduction and fertilization [91]. Thus, these unique features developed in sterile triploid *C. sativus* have evolved a large number of candidate loci that could be incorporated in further breeding program of desired varieties with improved quality and/or stronger resistance.

Previous studies on a list of sequenced plant genomes have shown that polyploidization was a prominent feature in the evolutionary history of angiosperms and that the WGD events, in particular, have made profound impacts on crop gene amplification and genome evolution [92], [93]. Here, we have identified 15,900 duplicated genes spanning 50.1% of the putative protein-coding genes in our transcriptomic data (Supplementary Table 10). Take advantage of these pairwise paralogs, we calculated an age distribution of synonymous substitution rates (*Ks*) that peaked at 0.36 and 1.48, providing clear evidence of two rounds of WGD events occurred at ~ 28 and ~ 114 Mya in *C. sativus* (Fig. 2D).

In particular, we further compared our transcriptomic data with other two genomic data of representative monocots (*A. officinalis* and *A. comosus*), based on the distribution of *Ks* values derived from their respective paralgous gene pairs. Our results confirmed that the ancient WGD event, referred as *τ* for monocots, was shared among *C. sativus*, *A. officinalis* [67] and *A. comosus* [66]. This *τ* event, thus, was considered to occur in the common ancestor of monocots. Another WGD event was found and designated as the recent WGD β) event in *C. sativus* (Fig. 2D). By contrast, there were two recent WGD events (namely α and β) occurred in *A. officinalis* and *A. comosus* (Fig. 2D). As the peaks of these recent WGD events separated from each other, the distinct β event in *C. sativus* was most likely to occur after divergence with *A. officinalis*. Upon the WGD event, new gene copies could undergo relaxed selections shortly after their duplication in the genome, which enables them to tolerate almost all nucleotide changes [94]. Subsequently, the WGD event would allow the survival of polyploidy in short-term and the formation of species in long-term [95]. In *C. sativus*, more than 35% duplicates have survived after the β WGD event. The robustness is therefore essential in the innovation of gene families associated with the regulatory and synthetic pathways of distinct secondary metabolites that are unique to *C. sativus*. However, our results are unable to conclude whether the β event was a species-specific or genus-specific WGD event due to lacking of genomic and genetic information in the genus *Crocus*.

### 3.3 Novel insights into the evolution of apocarotenoid biosynthetic pathway in C. sativus

The stigmas of *C. sativus* are used to make saffron and related products with distinct health-giving properties. Their qualities are highly dependent on three major secondary metabolites, i. e. crocin, picrocrocin and safranal. To gain novel insights into the molecular mechanism underlying the biosynthesis of apocarotenoids in *C. sativus*, we have performed an integrated analysis focusing on the major metabolic pathway. According to the annotation of enzyme-coding genes in our transcriptomic data, we obtained homologous members of structural genes potentially participated in the biosynthesis of apocarotenoids, including the nine genes (GPPS, GGPPS, PS, PDS, Z-ISO, ZDS, CrtISO, β-LYC and BCH) that catalyzed the general carotenoid biosynthesis, and the four genes (CCD, UGT, ALDH and β-GS) producing distinct apocarotenoids following the cleavage of carotenoids. Our analysis revealed that both the monocot and dicot species possess all of the structural genes (Supplementary Table 11). This confirmed that apocarotenoid biosynthetic pathway was appeared in the common ancestor of plants and retained for hundred million years [96].

**Fig. 3:**
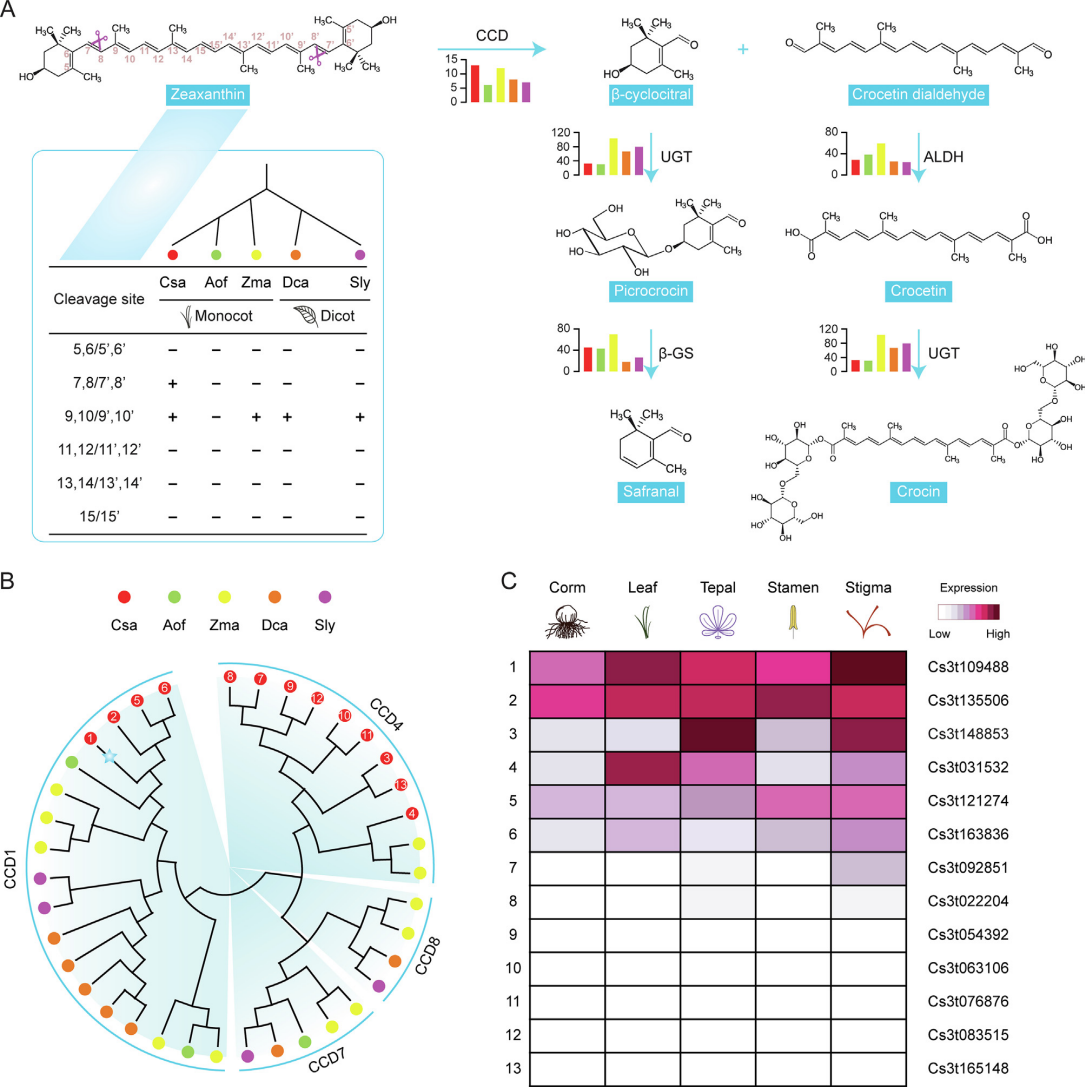
Evolutionary relationships and expression patterns of the key genes involved in apocarotenoid biosynthesis. (a) Biosynthetic pathway for producing distinct apocarotenoids through the cleavage of zeaxanthin. CCD, UGT, ALDH and β-GS represented gene-encoding enzymes of carotenoid cleavage dioxygenase, UDP-glucosyl transferase, aldehyde dehydrogenase and β-glucosidase, respectively. The histogram next to each enzyme showed the distribution of corresponding gene members identified from *C. sativus*, *A. officinalis*, *Z. mays*, *D. carota* and *S. lycopersicum*. Only the number of CCD members in *C. sativus* was relatively higher than other species. Table in the left box denoted zeaxanthin with different cleavage sites that were available by the CCD enzymes in the five representative species. Three-letter acronym for the abbreviation of each species name. (b) Neighbor-joining (NJ) phylogenetic tree of 38 CCD proteins constructed from the five representative plant species. Four subfamilies were grouped according to the substrate preference and cleavage specificity. In *C. sativus* (Csa, red solid dots), 13 CCD members were clustered into CCD1 and CCD4. The putative member for cleaving zeaxanthin at 7,8/7′,8′ double bonds was identified by similarity search against CsCCD2 and intended to be Cs3t109488 (blue pentagram). The numeric values within each red solid dot corresponded to the serial number given in the subgraph C. (c) Expression patterns of 13 CCD gene members (rows) from *C. sativus* based on the SGS RNA-Seq reads from five different tissues (columns). The heatmap was drawn with log_2_ transformation of gene expression data. (For interpretation of the references to color in this figure legend, the reader is referred to the web version of this article.)

Nevertheless, the composition and proportion of apocarotenoids varied largely across different plant species, thus responsible for their own distinctive physicochemical properties [97]. In fact, *C. sativus* holds a very special place chiefly because it is the only plant species that naturally produces crocin, safranal and picrocrocin in significant quantities. Biochemical analysis showed that crocin, safranal and picrocrocin is produced by cleaving zeaxanthin predominantly at the symmetric 7,8/7′,8′ double bonds in *C. sativus* [98]. In fact, the substrate zeaxanthin is usually converted into 3-hydroxy-β-ionone through the cleavage at the 9,10/9′,10′ double bonds in most plant species, such as *Z. mays* [99], *D. carota* [100], *S. lycopersicum* [101] as well as *C. sativus* [102] (Fig. 3A). Therefore, the cleavage specificity of zeaxanthin at the 7,8/7′,8′ positions suggests that a novel function of the CCD family has been independently evolved during the speciation of *C. sativus*.

To comprehensively investigate the evolutionary landscape of CCD family in *C. sativus*, we identified a total of 13 members belonging to the CCD family from our predicted protein-coding genes. This number is greater than those from *Z. mays* (12), *D. carota* (8), *S. lycopersicum* (7) and *A. officinalis* (6) (Fig. 3A; Supplementary Table 11). Using protein sequences, the neighbor-joining phylogenetic tree was constructed with a total of 38 CCDs from the five representative plant species. Phylogenetic analysis showed that these 38 CCD proteins could be apparently grouped into four subfamilies, designated as CCD1, CCD4, CCD7 and CCD8, according to their substrate preference and cleavage specificity (Fig. 3B). Among them, three were shared by the monocot and dicot species, further supporting that CCD family is extremely ancient. Meanwhile, CCD members from the same species tended to be grouped in the same cluster, revealing that a series of recent WGD events have occurred after species divergence. Theoretically, the expansion of CCD family has allowed one or more of them to evolve with novel functions.

In *C. sativus*, the key enzyme cleaving the 7,8/7′,8′ double bonds of zeaxanthin was recently identified and named as CsCCD2 (here is Cs3t109488) [19]. As shown in Fig. 3B, CsCCD2 was clustered within the CCD1 subfamily, suggesting that CsCCD2 has evolved from CCD1 and developed dedicated cleavage site after the divergence of *Crocus*. This is consistent with the findings previously reported [23]. Furthermore, we calculated the *Ks* value between CsCCD2 and its duplicate counterpart (Cs3t135506) in the present study. The value of 0.44 indicates that CsCCD2 was likely expanded from the recent β WGD event. After duplication, CsCCD2 might acquire an opportunity to gain a novel biological function upon selective pressures over successive generations of *C. sativus*. Undoubtedly, the favorable effects resulting from apocarotenoid cleavage products have endowed *C. sativus* with a competitive advantage in response to either environmental adaptation [103] or human demand [4], [11], [12], [13].

Gene expression profile analysis of the CCD gene members for five representative tissues (corm, leaf, tepal, stamen and stigma) showed that the CsCCD2 gene was constitutively expressed in all tissues with remarkably higher level (~5.77-fold on average, *P*-value < 0.001) in the stigma where apocarotenoids are biosynthesized and accumulated (Fig. 3C). This proved that the young member of CCD gene is indeed active and highly expressed in the stigma during the flowering stage of *C. sativus*. Meanwhile, the differentially expressed genes (DEGs) were identified via pair-wise comparisons of gene expression patterns between stigma and other four tissues (corm, leaf, tepal and stamen). The numbers of DEGs ranged from 87 to 1366 in different pair-wise comparisons, obviously showing transcriptional dynamics of genes among different tissues (Supplementary Table 12). But how *C. sativus* coordinates the expression of a novel protein-coding gene to divert the flux towards apocarotenoid biosynthesis in the right tissue at the right time needs to be elucidated in further analysis.

## 4 Conclusions

We present a high-quality SMRT sequencing datasets of full-length transcriptome for the *C. sativus*, whose genome sequence is not yet available. A total of 31,755 non-redundant sequences were captured, which could significantly improve the sequence integrity and functional annotation of putative protein-coding genes. Meanwhile, the obtained transcriptome may help partially clarifying the evolution history of *C. sativus* in general as well as secondary metabolite genes in particular. The key enzyme CCD2 involved in apocarotenoid biosynthesis was implicated to be evolved from the recent β WGD event. In addtion, our results will facilitate further genetic studies and crop improvement for *C. sativus*.

## 5 Accession number

The raw reads generated in this study have been deposited in the NCBI sequence read archive (SRA) under the accession number PRJNA542799 (http://www.ncbi.nlm.nih.gov/bioproject/PRJNA542799).

## CRediT authorship contribution statement

**Junyang Yue:** Conceptualization, Methodology, Writing - original draft, Writing - review & editing, Supervision. **Ran Wang:** Methodology. **Xiaojing Ma:** Supervision, Funding acquisition. **Jiayi Liu:** Resources. **Xiaohui Lu:** Resources. **Sambhaji Balaso Thakar:** Formal analysis. **Ning An:** Methodology. **Jia Liu:** Writing - review & editing. **Enhua Xia:** Methodology, Supervision. **Yongsheng Liu:** Writing - review & editing, Project administration, Funding acquisition.

## Declaration of Competing Interest

The authors declare that they have no known competing financial interests or personal relationships that could have appeared to influence the work reported in this paper.

## Appendix A Supplementary data

Supplementary data 1 Table S1. Statistics of sequence quality and annotation information for the SMRT data. Table S2. Categorization of 1130 putative genes within 64 transcription factor families. Table S3. Predicted target genes of the obtained miRNAs. Table S4. The enriched GO terms for the specific genes in *C. sativus*. Table S5. The enriched Pfam domain for the specific genes in *C. sativus*. Table S6. The enriched GO terms for the expanded genes in *C. sativus*. Table S7. The enriched Pfam domain for the expanded genes in *C. sativus*. Table S8. The enriched GO terms for the contracted genes in *C. sativus*. Table S9. The enriched Pfam domain for the contracted genes in *C. sativus*. Table S10. A total of 15,900 duplicated genes were identified in the SMRT data. Table S11. Number of structural genes potentially participated in apocarotenoid biosynthesis were identified from the five representative species. Table S12. Number of differentially expressed genes (DEGs) in different pair-wise comparisons.
